# Genomic analyses of human adenoviruses unravel novel recombinant genotypes associated with severe infections in pediatric patients

**DOI:** 10.1038/s41598-021-03445-y

**Published:** 2021-12-15

**Authors:** Joyce Odeke Akello, Richard Kamgang, Maria Teresa Barbani, Franziska Suter-Riniker, Christoph Aebi, Christian Beuret, Daniel H. Paris, Stephen L. Leib, Alban Ramette

**Affiliations:** 1 Institute for Infectious Diseases, University of Bern, Friedbühlstrasse 51, 3001 Bern, Switzerland; 2 Spiez Laboratory, Biology Division, Swiss Federal Office for Civil Protection, Spiez, Switzerland; 3 Graduate School for Cellular and Biomedical Sciences, University of Bern, Bern, Switzerland; 4 Department of Pediatrics, Bern University Hospital, Bern, Switzerland; 5 Swiss Tropical and Public Health Institute, Basel, Switzerland; 6 Department of Clinical Research, University of Basel, Basel, Switzerland

**Keywords:** Microbiology, Pathogens

## Abstract

Human adenoviruses (HAdVs) are highly contagious pathogens of clinical importance, especially among the pediatric population. Studies on comparative viral genomic analysis of cases associated with severe and mild infections due to HAdV are limited. Using whole-genome sequencing (WGS), we investigated whether there were any differences between circulating HAdV strains associated with severe infections (meningitis, sepsis, convulsion, sudden infant death syndrome, death, and hospitalization) and mild clinical presentations in pediatric patients hospitalized between the years 1998 and 2017 in a tertiary care hospital group in Bern, Switzerland covering a population base of approx. 2 million inhabitants. The HAdV species implicated in causing severe infections in this study included HAdV species C genotypes (HAdV1, HAdV2, and HAdV5). Clustering of the HAdV whole-genome sequences of the severe and mild cases did not show any differences except for one sample (isolated from a patient presenting with sepsis, meningitis, and hospitalization) that formed its own cluster with HAdV species C genotypes. This isolate showed intertypic recombination events involving four genotypes, had the highest homology to HAdV89 at complete genome level, but possessed the fiber gene of HAdV1, thereby representing a novel genotype of HAdV species C. The incidence of potential recombination events was higher in severe cases than in mild cases. Our findings confirm that recombination among HAdVs is important for molecular evolution and emergence of new strains. Therefore, further research on HAdVs, particularly among susceptible groups, is needed and continuous surveillance is required for public health preparedness including outbreak investigations.

## Introduction

Human adenoviruses (HAdVs) are pathogens of clinical importance and often used as model to understand biological systems^1^. As public health concern, HAdV infections are common and often cause acute diseases ranging from mild to severe, including death especially in immunocompromised individuals^2–6^. HAdVs are non-enveloped, double-stranded deoxyribonucleic acid (DNA) viruses belonging to the *Adenoviridae* family within the genus *Mastadenovirus*. At present, over 100 genotypes of HAdVs are recognized by the Human Adenovirus Working Group July, 2019 Update (http://hadvwg.gmu.edu/), which are classified into seven species/groups (HAdV-A to HAdV-G) initially defined on the basis of their physical, chemical and biological properties by serum neutralization methods, but more recently distinguished based on whole-genome sequencing (WGS), genomic and bioinformatic analysis (http://hadvwg.gmu.edu/).

The HAdVs that are predominately reported to be associated with human disease globally include genotypes from species C (HAdV1, HAdV2, and HAdV5), species B (HAdV3 and HAdV7), species E (HAdV4), and species F (HAdV41)^7–11^. In the pediatric population, HAdV infections are predominately caused by genotypes of the HAdV species C^10,12,13^. Although HAdV species C consists of only 6 genotypes so far (HAdV1, HAdV2, HAdV5, HAdV6, HAdV57 and HAdV89), these are reported to be more clinically significant than other HAdV species in causing severe infections with life-threatening implications in young children and immunocompromised patients^3,13^. More than half of HAdV infections in young children are associated with HAdV1 and HAdV2^2,10^. After primary infection, genotypes of HAdV-C species may establish latent infections and are capable of long-term persistence in lymphoid cells^14–16^. Thus, asymptomatic individuals can shed infectious viruses in stool for many years^17,18^.

HAdV genomes can be unstable as they are subjected to genetic drift resulting from base insertion, substitutions and deletions, but also are prone to changes observed as antigenic shifts originating from genomic recombination between at least two viral strains^19^. Early studies of HAdV recombination^20–22^ led to the hypothesis that molecular evolution of HAdV strains may be driven by recombination^19^. Moreover, recent studies have shown that recombination among circulating HAdV strains is frequent and plays a critical role in shaping the phylogenetic relationships among HAdV genomes^23^. To date, no recombinant HAdV has been reported in Switzerland. The aim of our study was to determine if there were any genomic differences between circulating HAdV strains causing severe and mild clinical presentations in hospitalized pediatric patients in Bern, Switzerland. We investigated the phylogenomic relationships and potential recombination events among Swiss HAdV isolates based on their whole-genome sequences. Overall, our results document that recombination is a major factor for HAdV evolution and may possibly contribute to disease severity.

## Results

### Genomic characteristics and comparative analysis

The complete genomes of HAdV isolates from pediatric patients presenting with severe cases including meningitis, sepsis, convulsion, sudden infant death syndrome, death, and hospitalization were analyzed alongside those obtained from HAdV pediatric patients presenting with mild cases (Table 1). The selection of mild cases for genomic comparative purposes was based on clustering of their partial hexon gene nucleotide sequences on the phylogenetic tree in comparison to partial hexon sequences isolated from severe cases. All patient isolates in this study were initially typed by Sanger sequencing of the hypervariable hexon region 1–7^10^. Molecular typing based on this region of a diagnostic specimen is sufficient to identify circulating HAdV genotypes causing infection among hospitalized patients. However, it does not allow for precise and accurate resolution among genotypes in particular identifying and assessing potential recombination events along with genome rearrangements. The HAdV genotypes analyzed in this study belonged to species C as these were the ones implicated in causing severe infection including death among the pediatric patients in our study collection. Of the initial 14 HAdV species C samples analyzed, three belonged to HAdV1, nine belonged to HAdV2 and two belonged to HAdV5 (Table 1). The genome length of samples identified as HAdV1 ranged from 35,858 to 35,974 bp, HAdV2 from 35,813 to 35,951 bp, and HAdV5 from 35,872 to 35,881 bp. The G+C content of genotypes belonging to HAdV species C (i.e., HAdV1, HAdV2 and HAdV5) varied between 55.20 and 55.40%. The percentage nucleotide identities between the complete genome sequences of the HAdV prototypes and strains of each genotype identified in this study was 97.58–98.97% among HAdV1 strains, 98.84–99.4% among HAdV2 strains, and 98.11–98.38% among HAdV5 strains. A slight variation in the percentage similarity at the nucleotide and amino acid level based on the three major capsid genes (penton-base, hexon, and fiber) of the HAdV1, HAdV2 and HAdV5 strains in this study was observed (Table 2).

**Table 1:** Characteristics of HAdV isolates in this study.

Isolate	Genotype (partial hexon)	Year of isolation	Patient’s age range	Patient’s sex	Specimen type	Clinical symptoms and outcome*	Genome size (bp)	Genotype classification (WGS)
ADVJA-00-BE	HAdV2	2006	1–5 year	F	Upper respiratory tract	Meningitis suspected, but not confirmed; no hospitalization	35,843	HAdV2
ADVJA-05-BE	HAdV1	2014	1–5 year	M	Lower respiratory tract	**Sudden infant death syndrome**	35,974	HAdV1
ADVJA-13-BE	HAdV1	2002	1–5 year	F	Upper respiratory tract	**Meningitis**	35,858	HAdV1
ADVJA-23-BE	HAdV2	2005	1–5 year	M	Lower respiratory tract	**Death**	35,813	HAdV2
ADVJA-38-BE	HAdV2	2010	< 1 year	M	Upper respiratory tract	**Fever, convulsions, hospitalization**	35,883	HAdV2
ADVJA-41-BE	HAdV1	2014	1–5 year	M	Stool	Gastroenteritis	35,859	HAdV1
ADVJA-54-BE	HAdV2	2006	1–5 year	F	Stool	Abdominal pain, fever, gastroenteritis, no therapy	35,826	HAdV2
ADVJA-56-BE	HAdV2	2006	1–5 year	F	Stool	Gastroenteritis	35,884	HAdV2
ADVJA-60-BE	HAdV2	2000	< 1 year	M	Stool	**Gastroenteritis, hospitalization**	35,839	HAdV2
ADVJA-65-BE	HAdV5	2013	1–5 year	F	Stool	Oncological immunosuppressed, gastroenteritis	35,881	HAdV5
ADVJA-77-BE	HAdV2	2009	< 1 year	M	Upper respiratory tract	Cough, upper respiratory infection	35,951	HAdV2
ADVJA-78-BE	HAdV2	2006	1–5 year	M	lower respiratory tract	**Sudden infant death syndrome**	35,890	HAdV2
ADVJA-749-BE	HAdV2	2002	1–5 year	M	upper respiratory tract	**Sepsis, meningitis, hospitalization**	35,853	HAdV-C
ADVJA-949-BE	HAdV5	2012	< 1 year	F	lower respiratory tract	**Sudden infant death syndrome**	35,872	HAdV5

*Clinical symptoms indicated in bold are severe cases of HAdV infection.

**Table 2:** Percentage (%) similarity at nucleotide and amino acid levels of HAdV1, HAdV2, and HAdV5 strains in this study.

Region	% Similarity at	HAdV1 (N = 3)	HAdV2 (N = 9)	HAdV5 (N = 2)
Penton	Nucleotide	98.90–99.94	99.36–100	97.80
	Amino acid	98.61–99.83	99.47–100	98.26
Hexon	Nucleotide	99.79–99.97	98.69–100	96.75
	Amino acid	100	99.79–100	98.74
Fiber	Nucleotide	99.20–99.66	99.71–100	98.17
	Amino acid	98.80–99.14	99.66–100	97.76

The penton base, hexon and fiber were chosen because they play an important role in determining tissue tropism.

### Phylogenetic analysis

**Figure 1:**
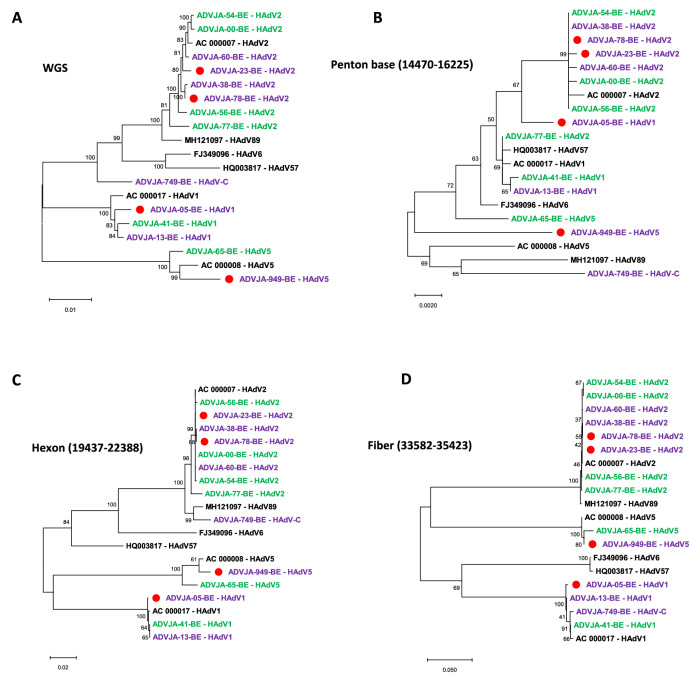
Phylogenetic analysis of HAdV genotypes identified from severe and mild cases in pediatric patients based on complete genome sequences (**A**), complete penton base sequences (**B**), complete hexon gene sequences (**C**), and complete fiber gene sequences (**D**). Eight severe cases (highlighted in purple, of those who died indicated with a red circle), six mild cases (highlighted in green), and GenBank sequences of the prototype strains are indicated in bold black font. Labelling indicates isolate names or accession number (for prototype strains), followed by HAdV genotype/species classification. Alignment positions relative to prototype sequence AC_000007 are indicated for each major HAdV gene (penton base, hexon, and fiber). Phylogenetic trees were generated using the Maximum Likelihood method based on Jukes-Cantor model^24^ with 1000 bootstrap replicates. The percentage of trees in which the associated taxa clustered together is shown next to the branches. The tree is drawn to scale, with branch lengths proportional to the number of substitutions per site.

Phylogenetic analysis was performed on whole-genome sequences and on complete sequences of penton base, hexon and fiber genes to investigate the genetic relationships between the Swiss HAdV strains and the prototype HAdV strains obtained from GenBank (Fig. 1). Whole-genome phylogenetic analysis of the eight severe and six mild cases did not show any differences except for ADVJA-749-BE (isolated from a patient presenting with sepsis, meningitis and was hospitalized) that formed a separate cluster among other HAdV species C genotypes (Fig. 1A). Overall, clustering of the complete hexon and fiber gene sequences agreed with clustering of the complete genomic sequences except for isolate ADVJA-749-BE with intertypic recombination affecting the hexon and fiber gene (Fig. 1A,C,D). Phylogenetic analyses of the penton base gene showed a completely different clustering with relatively low bootstrap support values (Fig. 1B). This suggests high identity within the penton base gene between genotypes of the same HAdV species. Phylogenetic analysis of the three HAdV coding regions (penton base, hexon, and fiber) demonstrated that ADVJA-749-BE strain exhibited a close relationship to HAdV89 in its penton base, and hexon gene, and to HAdV1 in its fiber gene (Fig. 1B–D, respectively).

### Comparative genomic analyses of a potentially “novel” HAdV-C genotype

**Figure 2:**
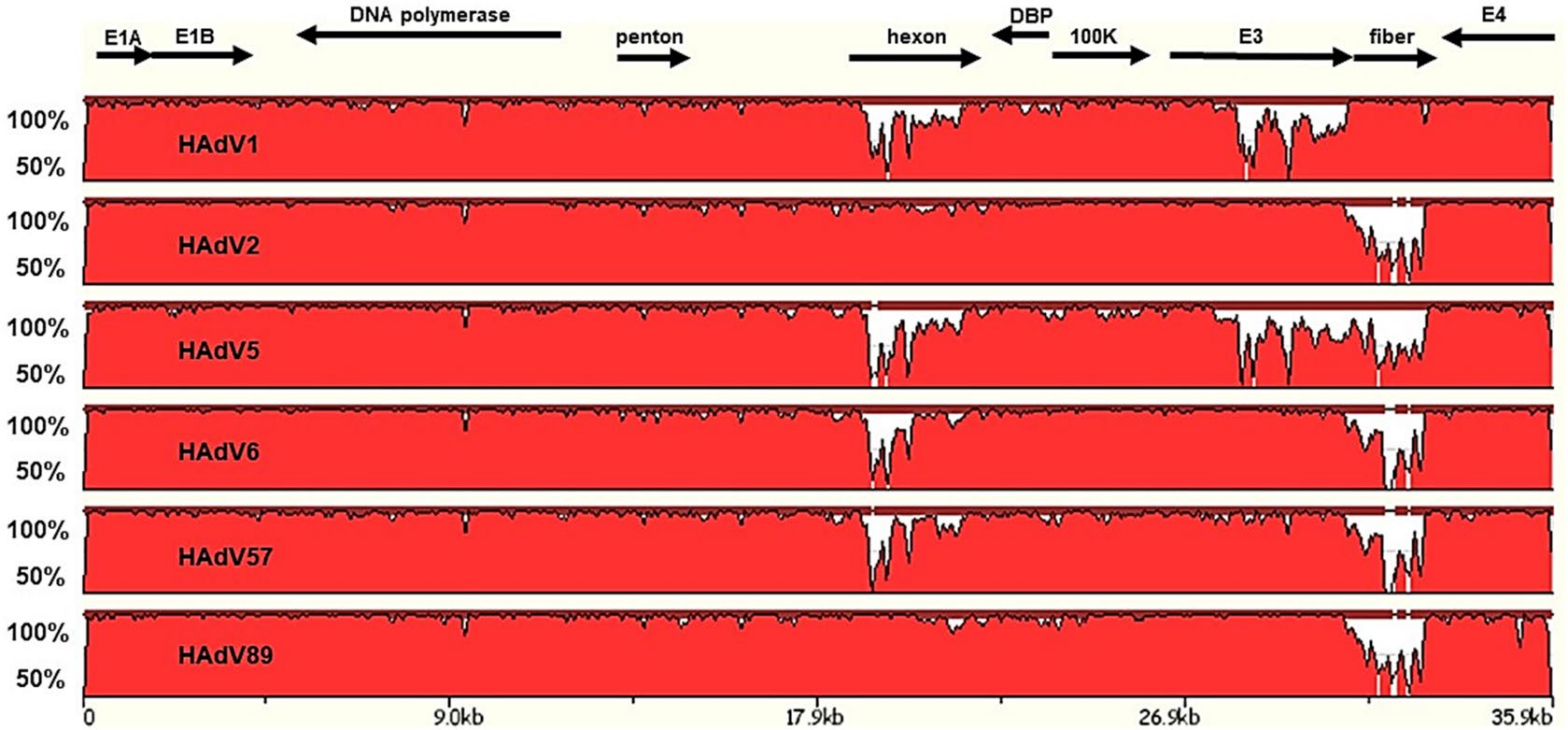
Pairwise genome comparative analysis of HAdV species C strains. zPicture (https://zpicture.dcode.org/) analysis based on BlastZ local alignment algorithm to show the regions of nucleotide sequence similarity between ADVJA-749-BE strain (used as query) and the representative prototype strains of HAdV species C. The Y-scale represents the percentage identity of genome pairs from 50 and 100%. Arrows indicate approximate positions of the coding transcripts and their orientation.

To determine the genomic characterization of ADVJA-749-BE strain, comparison with all prototype strains of HAdV species C was performed. Compared with the complete HAdV species C sequences of the 6 prototype strains of HAdV1 (AC_000017.1), HAdV2 (AC_000007.1), HAdV5 (AC_000008), HAdV6 (FJ349096), HAdV57 (HQ003817), and HAdV89 (MH121097), ADVJA-749-BE strain shares the highest nucleotide similarity (97.37%) at the full genome level with the prototype strain HAdV89 (Table 3). Highest similarity with this prototype strain was also observed within the penton base (98.31%), hexon (98.01%), E1B 55K (99.73%) and the E3 ORF (99.58%). The latter region of ADVJA-749-BE also had the same nucleotide identity (99.58%) with the prototype strain of HAdV2 and HAdV6. The prototype strains HAdV2 and HAdV6 also showed the greatest similarities to ADVJA-749-BE in the E1A (99.89%). Furthermore, prototype strain HAdV2 showed the highest similarities with ADVJA-749-BE within the DBP (99.06%) and 100K (99.63%) genes. Within the DNA polymerase gene, ADVJA-749-BE shared highest similarity (99.67%) to HAdV6. On the other hand, the ADVJA-749-BE strain shared higher identities with HAdV1 strain in the fiber region (Table 3). A high level of similarity and identity across the genomes of HAdV species C was observed (Fig. 2). The results obtained complement the phylogenetic analysis demonstrating that the ADVJA-749-BE strain is highly similar to the prototype strains of HAdV species C but with differences across the entire genome particularly showing diversity in the hexon, E3 and fiber (Fig. 2).

**Table 3:** Percentage nucleotide (nt) sequence identities between ADVJA-749-BE and representative prototype HAdV species C genotypes.

Region	% nt identity					
	ADVJA-749-BE strain					
	HAdV1 (%)	HAdV2 (%)	HAdV5 (%)	HAdV6 (%)	HAdV57 (%)	HAdV89 (%)
E1A	98.28	**99.89**	99.31	**99.89**	99.77	99.20
E1B 55k	97.80	99.66	97.93	99.60	98.00	**99.73**
DNA polymerase	98.64	99.30	99.30	**99.67**	98.64	99.33
Penton base	97.33	97.73	98.14	97.45	97.33	**98.31**
Hexon	85.12	97.39	81.98	87.55	86.76	**98.01**
DBP	96.60	**99.06**	97.30	98.81	97.36	97.55
100K	98.23	**99.63**	96.83	99.38	97.98	98.64
Fiber	**99.31**	71.95	74.60	70.38	70.27	71.89
E3 ORF	87.71	**99.58**	86.04	**99.58**	97.29	**99.58**
E4 ORF	99.55	**99.77**	99.32	99.10	97.74	98.87
Full genome	95.29	97.31	93.54	96.37	95.50	**97.37**

Highest values are indicated in bold.

### Genomic recombination analysis

**Figure 3:**
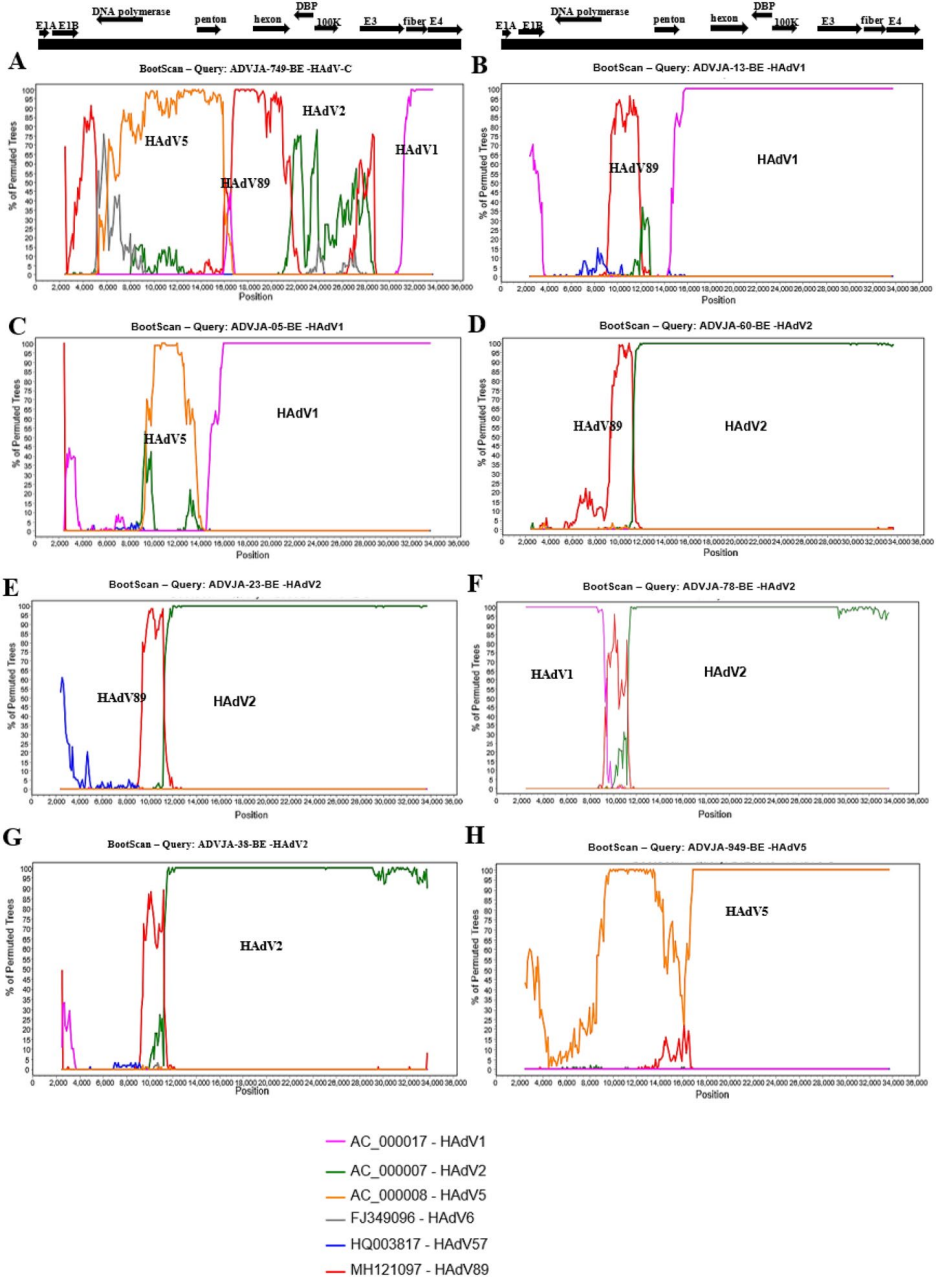
Bootscan analysis of the whole-genome sequences for severe HAdV isolates compared with sequences of prototype HAdV1, HAdV2, HAdV5, HAdV6, HAdV57, and HAdV89. Bootscan of whole-genome sequence from patient presenting with sepsis, meningitis and was hospitalized due to HAdV species C genotype (**A**), meningitis due to HAdV1 (**B**), sudden infant death syndrome due to HAdV1 (**C**), gastroenteritis and hospitalization due to HAdV2 (**D**), death resulting from HAdV2 (**E**), sudden infant death syndrome due to HAdV2 (**F**), fever, convulsion, and hospitalization due to HAdV2 (**G**), and sudden infant death syndrome due to HAdV5 (**H**). Analyses were performed using SimPlot (see “Methods” section). The genotypes involved in recombination events for each of the severe cases are indicated on each panel. The black bar at the top represents the genome map with black arrows indicating approximate position of the coding transcripts and their direction. The legend shows the representative prototype HAdV species C strains used for comparison with the labelling as accession number—HAdV genotype. The percentage of permutated trees that supported grouping are marked along the y-axis and the genome nucleotide position are indicated along the x-axis. Parameter setting for the recombination analysis using Bootscan in the Simplot software were: window size (5000 nucleotides), step size (100 nucleotides), replicates used (n = 100), gap stripping (on), distance model (Kimura) and tree model (Neighbor-joining).

To investigate potential recombination events within and between HAdV genomes isolated in this study, particularly the genome sequences isolated from HAdV severe cases, we performed Bootscan analysis with studied Swiss strains and representative HAdV species C prototype strains available in GenBank (Fig. 3). Of note was isolate ADVJA-749-BE which was associated with multiple recombination events. The genome sequence of ADVJA-749-BE that was isolated from a pediatric patient presenting with sepsis, meningitis and hospitalized was identified as a possible recombinant that may have arisen from recombination events involving HAdV1, HAdV2, HAdV5, and HAdV89. Unlike this potentially new recombinant isolate ADVJA-749-BE, which has HAdV5 penton base, HAdV2/HAdV8 hexon and HAdV1 fiber, the hexon and fiber sequences of HAdV2 and HAdV89 were similar. The other samples from severe and mild cases also showed some potential recombination events, but to a lesser extent (Fig. 3, Supplementary Fig. S1). For severe infections due to HAdV species C, 75% (6 out of 8 cases) had potential recombination events involving at least two of the representative HAdV species C prototype strains, while 66.7% (4 out of 6 cases) of the mild infections had potential recombination events involving also at least two of the HAdV species C prototype strains.

### Amino acid analysis of the potentially “novel” HAdV-C genotype with parent prototype strains

**Figure 4:**
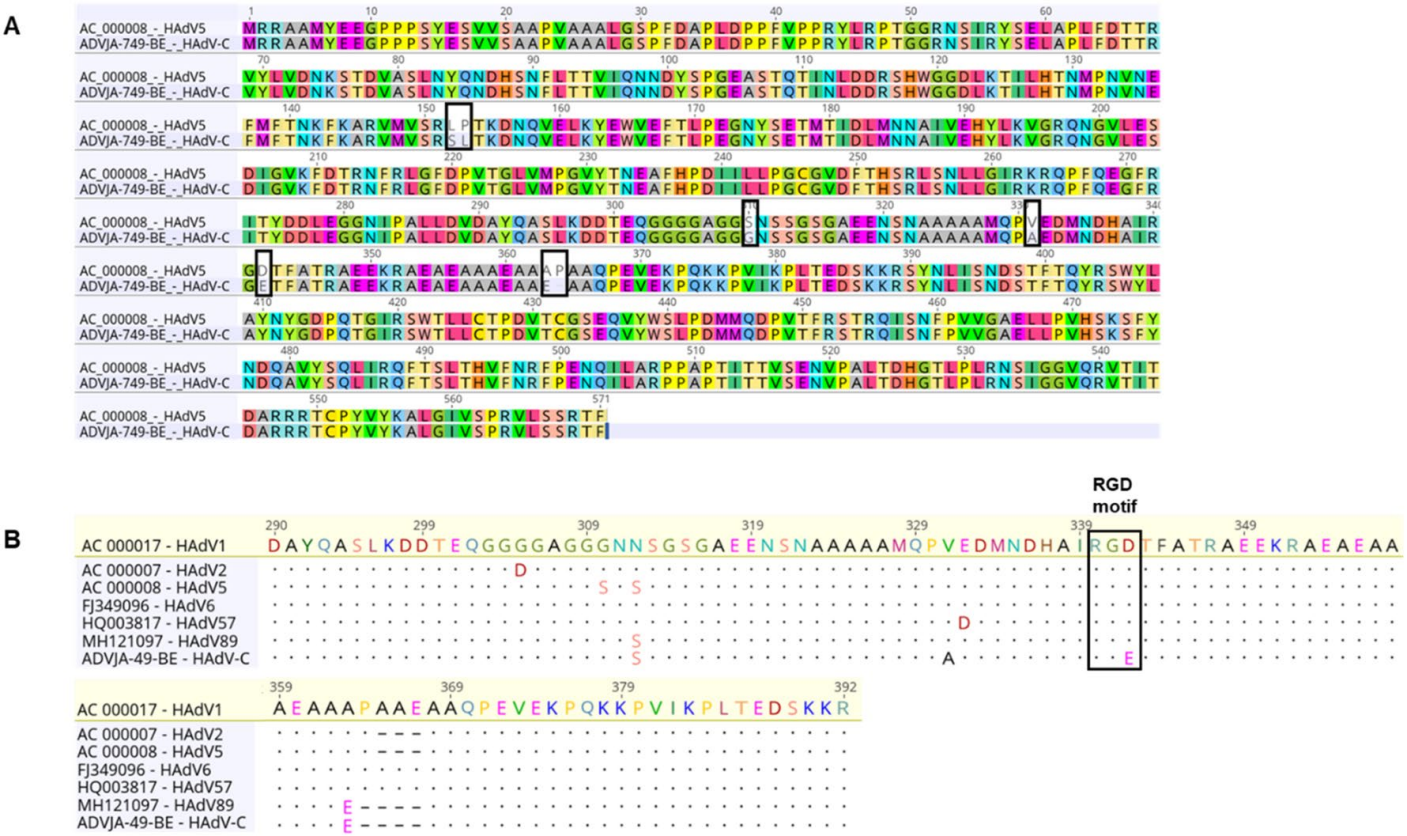
Amino acid sequence alignments of the penton base. (**A**) Pairwise alignment of the potentially novel HAdV-C (ADVJA-749-BE) with the HAdV5 prototype strain (AC_000008). (**B**) RGD loop sequence of the penton base for all the HAdV C prototype sequences strains with the potentially novel HAdV-C (ADVJA-749-BE) genotype. Alignment visualized with Geneious (see “Methods” section).

To assess the overall sequence similarity of the novel HAdV C (ADVJA-759-BE) with its candidate parent HAdV C prototype sequence strains (HAdV5 penton, HAdV2/89 hexon and HAdV1 fiber), an amino acid alignment was performed (Fig. 4). Compared with the HAdV5 prototype strain (AC_000008), the novel HAdV C (ADVJA-759-BE) had 98.8% identical sites, a pairwise identity of 98.8% with the complete penton base, two amino acid substitutions (L152S and P153L) in the hypervariable region 1 (HVR1) of the penton base, four amino acid substitutions (S310G, V331A, D342E and A363E) and one amino acid deletion (P364) in the RGD loop of the penton base (Fig. 4A). Although the amino acid substitution (A363E) and deletion (P364) were shown to be unique to HAdV89 by Dhingra et al.^25^, we found that the novel HAdV C (ADVJA-759-BE) also has the substitution (A363E) and deletion (P364) in the RGD loop of the penton base. Compared with other HAdV C prototype sequences strains, ADVJA-759-BE had one unique amino acid substitutions (V331A) in the RGD loop and one unique substitution (D342E) in the RGD motif region of the penton base (Fig. 4B). Interaction of the penton base RGD motifs with cellular integrins facilitates virus internalization^26^.

There were 99.2% identical protein sites among HAdV2, HAdV89 and the novel HAdV C (ADVJA-759-BE) and pairwise identity of 99.4%. Compared with the HAdV2 prototype strain (AC_000007.1), the novel HAdV C (ADVJA-759-BE) had one amino acid addition (149E), one amino acid substitution (E200G) in the HVR1-6 region of the hexon and three amino acid substitutions (G448D, S449A and D454N) in the HVR7 region of the hexon. Compared with the HAdV89 prototype strain (MH121097), the novel HAdV C (ADVJA-759-BE) had two amino acid substitutions (E200G and L306M) in the HVR1-6 region of the hexon protein and one substitution (G448D) in the HVR7 region of the hexon protein (Fig. S2). Compared with the HAdV1 prototype strain (AC_000017.1), the novel HAdV C (ADVJA-759-BE) had 98.8% identical sites and 98.8% pairwise identity with the complete penton base and seven amino acid substitutions (K74E, N199S, H414Y, R442K, R510T, T563S and I565M) in the fiber protein (Fig. S3).

## Discussion

We conducted a comparative genomic analysis using whole-genome sequences of HAdV species C from hospitalized pediatric patients in Bern, Switzerland. Among the six genotypes of HAdV species C, HAdV1 and HAdV2 were previously reported to cause higher morbidity than other genotypes among the pediatric patients hospitalized between the year 1998 and 2017 in a tertiary care hospital group in Bern, Switzerland covering a population base of 2 million inhabitants^10^. A trend toward more severe cases due to HAdV species C has also been reported by Esposito et al.^27^. To date, no recombinant HAdV has been reported or identified in Switzerland, mainly because of a lack of studies on HAdV epidemiology and molecular evolution. Typing systems based on the partial gene sequence of one or more of the three major capsid genes (penton base, hexon, and fiber) are sufficient for gaining insight into the epidemiology of circulating HAdV strains. However, as homologous recombination of HAdV capsid genes plays a central role in shaping the evolution of HAdVs^25,28,29^ and may have consequences for HAdV detection and pathogenicity, information obtained on single gene sequences may not provide enough molecular resolution. Therefore, whole-genome sequencing based methods are recommended to investigate adenovirus phylogenomic relationships.

By employing whole-genome sequencing approach, fourteen HAdV-C whole-genome sequences (eight from severe cases and six from mild cases) were generated from hospitalized pediatric patients. The sequences were subjected to phylogenetic analyses and probed for potential recombination events and genome rearrangements, which are recognized as important mechanisms that may influence tissue tropism, and potentially pathogenicity and virulence of novel HAdV pathogens^25,29,30^. Global pairwise genome comparisons were also performed (Fig. 2). Our results demonstrated that the phylogenetic analysis of the complete genomes, hexon and the fiber genes in this study provided similar information regarding clustering of the HAdV strains except for one strain (ADVJA-749-BE) showing intertypic recombination affecting the hexon and fiber genes. The penton base phylogeny, however, failed to provide much meaningful information on HAdV species C strains as the 14 sequences did not show much divergence between each other (Fig. 1B), a result supported by the findings of Zhang and Huang^31^. Moreover, a study by Robinson et al. found that HAdV species C and E have similar penton base genes but show diversity in the hexon, fiber, and E3 ORFs^28^, which is also observed in our study (Fig. 2).

Of particular note was the identification of a strain (ADVJA-749-BE) that represents a potentially novel HAdV species C genotype isolated from a child presenting with sepsis, meningitis, and hospitalization, thus may be an etiological agent associated with sepsis and meningitis. Although the initial typing results based on the partial hexon gene identified HAdV2 as the cause of infection, phylogenomic analysis indicated that this strain formed a separate cluster among the other genotypes of HAdV species C (Fig. 1A), sharing the highest nucleotide identity (97.4% and 97.3%) with HAdV89 and HAdV2, respectively at the genome level (Table 3), but possessed fiber gene sequence identical to that of HAdV1 (Figs. 1D, 2, Table 3). Moreover, recombination analysis further confirmed that the ADVJA-749-BE strain was a recombinant of HAdV1, HAdV2, HAdV5, and HAdV89 (Fig. 3A). In addition, the amino acid sequence alignment of the novel HAdV-C versus the suggested parent (HAdV5 penton base, HAdV2/HAdV89 hexon and HAdV1 fiber) amino acid sequences showed a high similarity (Fig. 4)

Novel HAdV pathogens are well-known to arise from recombination events occurring only between HAdV genotypes of the same species and in regions of high sequence homology^20,21^. We therefore propose that ADVJA-749-BE isolate is a potentially novel genotype of HAdV species C, which may be an etiological agent associated with sepsis. Of the HAdV species C genotypes, HAdV57 (isolated from stool of a healthy child in 2001)^32^ and HAdV89 (identified from stool of an immunosuppressed patient in 2015)^25^ were both identified as recombinant viruses, with HAdV57 having a similar fiber gene to HAdV6 and harboring a unique hexon distinguished by its loop-2 motif^32^, whilst HAdV89 had a novel penton base sequence^25^. The circulation of recombinant HAdV species C strains has also been recently reported^31^.

For natural recombination to occur, co-infection is required. Co-infection by two or more genotypes of HAdV species has been documented^33^. A study by Lukashev et al., in which they analyzed 16 HAdV species C field strains at four genomic regions including the hexon, fiber, polymerase and E1A regions, suggested that recombination is frequent^23^. This finding was also supported by a recent study by Dhingra et al. which demonstrated that potential multiple recombination events within the E1 and E4 gene regions are likely to contribute to the evolution of species HAdV-C^25^.

Overall, recombination and genomic rearrangements within the three major HAdV capsid genes (penton base, hexon, fiber) that are important determinants of tropism, as well as the E3 region that harbors genes affecting the host immunity after virus infection^34^ may contribute to disease severity. Nevertheless, further work is needed to verify factors contributing to HAdV disease severity so as to better understand ways of developing effective preventive and therapeutic measures.

## Methods

### Samples

This study was based on already stored material at the Institute for Infectious Diseases (IFIK). There was no intervention or interference with patient management, as clinical samples were already obtained routinely at IFIK for viral diagnostics. Decision to send samples to IFIK remained at the sole discretion of the physician in charge of the patients.

We defined severe cases retrospectively as those associated with death or requiring hospitalization. HAdV isolates from patients presenting with severe cases and a subset of selected mild cases were obtained from IFIK clinical bio-bank. Selection criteria of HAdV isolates associated with mild cases was based on HAdV hexon sequences that phylogenetically clustered in the same as or different branches than the HAdV severe cases (Fig. 1), as determined previously^10^. This study was approved by the Swiss Ethics Committees on Research involving humans (BASEC-Nr:Req-2018-00158) which waived the requirement to provide informed consent given that the study was retrospective, based on already stored material obtained for the same, original viral diagnostic purposes, and did not interfere with patient management or treatment. The study was conducted in accordance with the present protocol, the current version of the “Declaration of Helsinki”, the “Good Clinical Practice (GCP)” Guidelines, Swiss law and the requirements of the competent authorities and the Ethics Committee.

### Cell lines and virus DNA isolation

Cell cultures were performed in A549 cells purchased from the American Type Culture Collection (Manassas, VA, USA) and were maintained in Earle’s minimal essential medium (MEM) (Biochrom GmbH, Germany) supplemented with 2.2 g/l NaHCO_3_ (Biochrom), 1% l-glutamine (Merck, Germany), 1% penicillin (10,000 U/ml) and streptomycin (10,000 μg/ml) (Biochrom), 1% fungizol (CPS Cito Pharma Services, Switzerland), and 1% heat inactivated Fetal Bovine Serum (FBS) (Biochrom) at 37 °C and 5% CO_2_. The samples inoculated in A549 cells were incubated at 37 °C for 3–7 days or until a cytopathic effect was observed. After cytopathic effect was confirmed, the positive cell culture supernatant was subjected to DNA extraction. DNA viral extraction from 200 µl supernatant of the cell cultured HAdV positive samples was automatically performed using the NUCLISENS easyMAG (bioMérieux, Geneva, Switzerland) extractor, as per manufacturer’s instructions. After DNA extraction, the DNA concentration of each sample was measured using the Qubit dsDNA high sensitivity assay kit on the Qubit 3.0 Fluorometer (ThermoFisher Scientific, Zug, Switzerland) as per manufacturer's protocol prior to whole genome amplification (WGA).

### Whole-genome amplification (WGA), preparation of sequencing libraries

The Seqplex enhanced DNA amplification kit (Sigma-Aldrich Chemie GmbH, Buchs SG, Switzerland) was used for WGA of the DNA as per manufacture’s protocol. The DNA was sheared to 400 bp using the Covaris M220 system (Covaris Ltd, Brighton, United Kingdom) prior to WGA amplification. The Covaris program for shearing was as follows; temperature 20 °C, duty factor 20, cycles/burst 200, peak power 50, and time 60 s. Libraries were constructed with the Ion plus fragment library kit using the ABI library builder according to the manufacturer’s instructions (ThermoFisher Scientific).

Sequencing barcoded libraries were prepared automatically on the ABI Library Builder System with the Ion plus Fragment Library Kit (ThermoFisher Scientific) according to the Ion Xpress Plus and Ion Plus Library preparation (ThermoFisher Scientific). The generated adapter-ligated libraries were subjected to size selection with 0.55× Agencourt AMPure XP magnetic beads (Beckman Coulter, Nyon, Switzerland), followed by measurement of the concentration using Qubit dsDNA High Sensitivity Assay kit (ThermoFisher Scientific) and assessment of the size distribution using the Agilent 2100 Bioanalyzer system with the Agilent High Sensitivity DNA kit (Agilent Technologies AG, Basel, Switzerland). Sample libraries were pooled and loaded automatically on the 530-chip using the Ion Chef instrument according to the manufacturer’s instructions. The loaded chip was then inserted into the Ion S5XL for sequencing with 850 flows using the Ion 530 (400 bp) chip kit.

### Bioinformatic analyses

Raw sequence data in BAM file format were imported into CLC genomics workbench v12.0.3 (QIAGEN, Aarhus, Denmark) and trimmed with the following parameters: Removal of adaptor/barcode sequences from both ends, discarding of reads with ambiguous bases, base quality below Q30, and read length below 50 nt. The trimmed reads were exported as fasta file from CLC genomics workbench and imported into Geneious Prime 2020.1.2 software (http://www.geneious.com^35^) for further analysis: The trimmed reads were normalized to 100× coverage using BBNorm (version 38.37) and duplicate reads were removed using Dedupe (version 38.37; k-mer seed length of 31). Reads were subjected to de novo assembly using SPAdes assembler (version 3.13.0) using default parameters. As the genotypes of most samples were previously identified based on Sanger sequencing of the partial hexon gene, the resulting contigs for each sample were mapped to HAdV prototype strain sequences of either HAdV1 (AC-000017), HAdV2 (AC-000007) or HAdV5 (AC-000008) to which it belonged using Bowtie2 (version 2.3.0), with “end-to-end” alignment and default parameters. Consensus sequences were produced and visually inspected. Following visual inspection of consensus sequences, gaps indicating missing nucleotides were identified and corrected by Sanger sequencing. Annotation of the consensus sequences was performed based on HAdV reference prototype strain genome annotations, using the Annotate & Predict tool within Geneious prime^35^. The annotations were manually checked and edited. The genome sequences for all 14 isolates were deposited to the European Nucleotide Archive, under project reference PRJEB40708.

### Phylogenetic and computational analysis

Multiple sequence alignments of HAdV sequences from patients along with HAdV reference prototype sequences for construction of phylogenetic trees were performed using MAFFT v7.450 (within Geneious Prime) with the default gap parameters. For detailed comparisons, sequences of prototype strains HAdV1 (AC-000017), HAdV2 (AC-000007), HAdV5 (AC-000008), HAdV6 (FJ349096), HAdV57 (HQ003817) and HAdV89 (MH121097) belonging to group HAdV-C obtained from GenBank were used for phylogenetic and genetic recombination analysis. Phylogenetic analysis was conducted in MEGAX (version 10.0.3^36^) using Maximum Likelihood method and Jukes-Cantor model^24^ with 1000 bootstrap replications. Bootscan analyses to identify potential recombination events were performed using SimPlot (version 3.5.1; https://sray.med.som.jhmi.edu/SCRoftware/simplot/) with parameters consisting of window size of 5000 bp, step size of 100 bp, gap stripping, 100 replicates, kimura (2-parameter), and Neighbor-Joining method. Global pairwise genome comparisons were performed using zPicture (https://zpicture.dcode.org/^37^).

## Supplementary Information

Supplementary Figures.
