# Supplementary material for: Genomic analyses of human adenoviruses unravel novel recombinant genotypes associated with severe infections in pediatric patients
Source: Sci Rep. 2021 Dec 15;11:24038. doi: 10.1038/s41598-021-03445-y (PMC8674331; doi:10.1038/s41598-021-03445-y)
Supplement: Supplementary file 1 — Supplementary Figures. [file 41598_2021_3445_MOESM1_ESM.docx]

**Supplementary material**

**Genomic analyses of human adenoviruses unravel novel recombinant genotypes associated with severe infections in pediatric patients**

Joyce Odeke Akello,^1– 3^Richard Kamgang,^1^ Maria Teresa Barbani,^1^ Franziska Suter-Riniker,^1^ Christoph Aebi ^4^, Christian Beuret, ^2^ Daniel H. Paris, ^5,6^ Stephen L Leib,^1^ Alban Ramette^1^

^1^Institute for Infectious Diseases, University of Bern, Bern, Switzerland

^2^Biology Division, Spiez Laboratory, Swiss Federal Office for Civil Protection, Spiez, Switzerland

^3^Graduate School for Cellular and Biomedical Sciences, University of Bern, Bern, Switzerland

^4^Bern University Hospital, University of Bern, Bern, Switzerland

^5^Swiss Tropical and Public Health Institute, Basel, Switzerland

^6^Department of Clinical Research, University of Basel, Basel, Switzerland

Correspondence: Alban Ramette. Institute for Infectious Diseases, University of Bern, Friedbühlstrasse 51, Bern 3001, Switzerland. Tel +41 31 632 9540, Email [alban.ramette@ifik.unibe.ch](mailto:alban.ramette@ifik.unibe.ch).


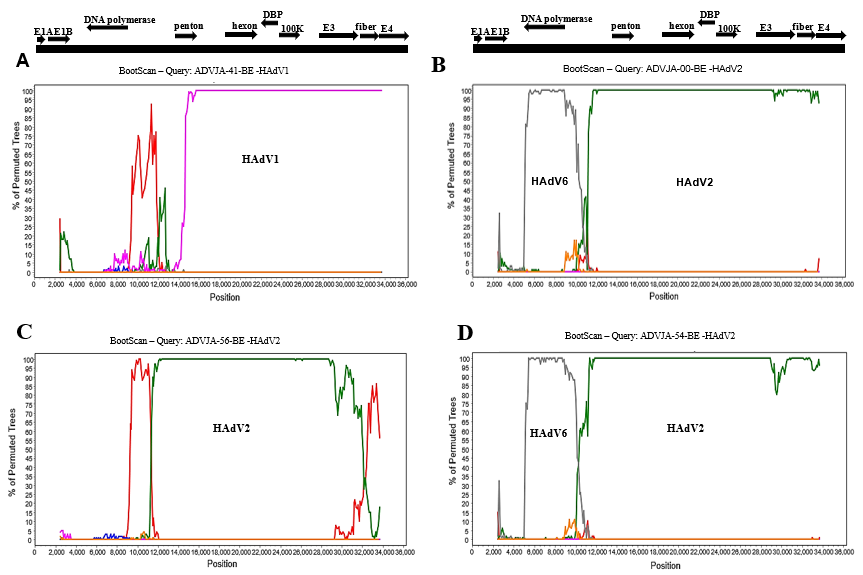

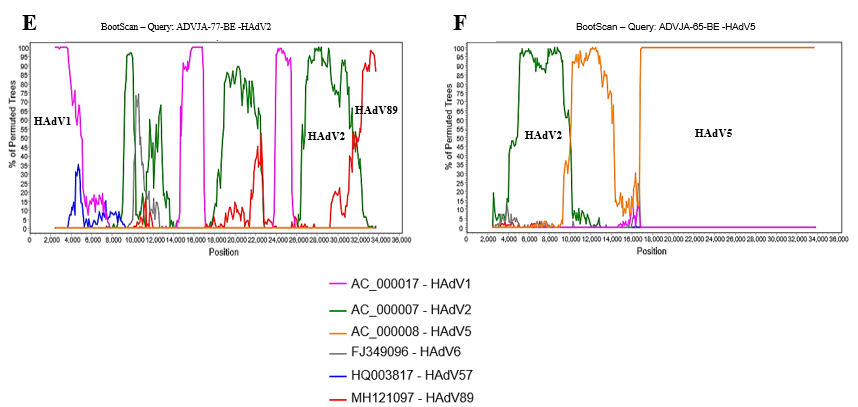


**Figure S1**. Bootscan analysis of the whole-genome sequences obtained from mild HAdV cases compared with the prototype sequences of HAdV1, HAdV2, HAdV5, HAdV6, HAdV57, and HAdV89. Bootscan of whole-genome sequence from patient presenting with gastroenteritis due to HAdV1 (A), meningitis suspected but not confirmed and no hospitalization due to HAdV2 (B), gastroenteritis due to HAdV2 (C), abdominal pain, fever, gastroenteritis and no therapy due to HAdV2 (D), cough and upper respiratory infection due to HAdV2 (E), oncological, immunosuppressed, and gastroenteritis due to HAdV5 (F).The genotypes involved in recombination events for each of the mild cases are indicated on each panel. The black bar at the top represents the genome map with the black arrows indicating approximate position of the coding transcripts and their direction. The legend shows the representative prototype HAdV species C strains used for comparison with the labelling as accession number – HAdV genotype. The percentage of permutated trees that supported grouping are marked along the y-axis and the genome nucleotide position are indicated along the x-axis. Parameter setting for the recombination analysis using Bootscan in the Simplot software were: window size (5000 nucleotides), step size (100 nucleotides), replicates used (n =100), gap stripping (on), distance model (Kimura) and tree model (Neighbor-joining).


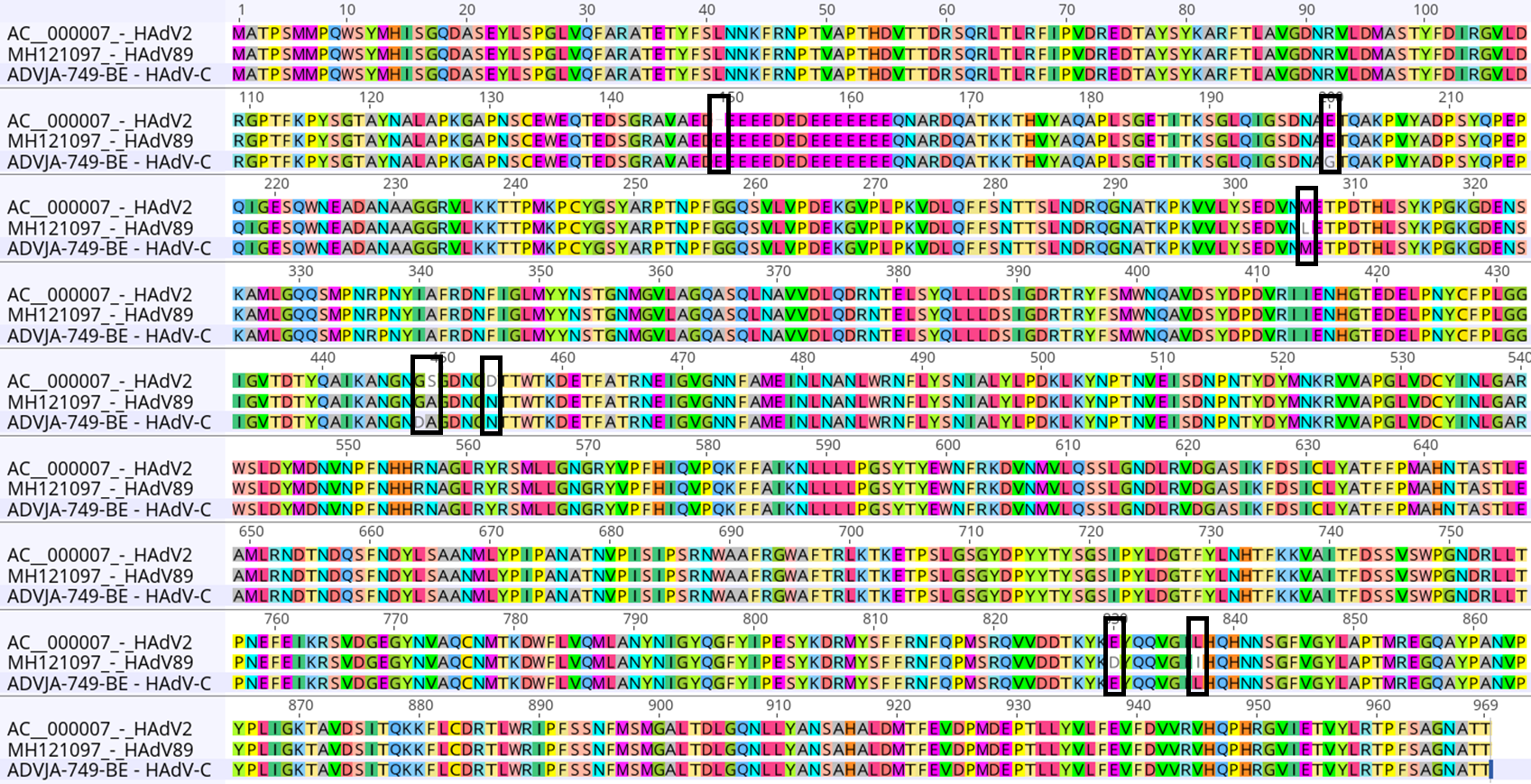


**Figure S2**. Multiple amino acid alignment of the potentially novel HAdV-C (ADVJA-749-BE) with the suggested parent hexon prototype sequence strains HAdV2 (AC_000007.1) and HAdV89 (MH121097). The name of the HAdV sequences used in the alignment analysis is indicated on the left-hand side of the figure. Labelling indicates isolate names or accession number (for prototype strains), followed by HAdV genotype/species classification. The black rectangle boxes indicated positions of amino acid differences between the potentially novel HAdV-C (ADVJA-749-BE) and the parent hexon prototype sequence strains.


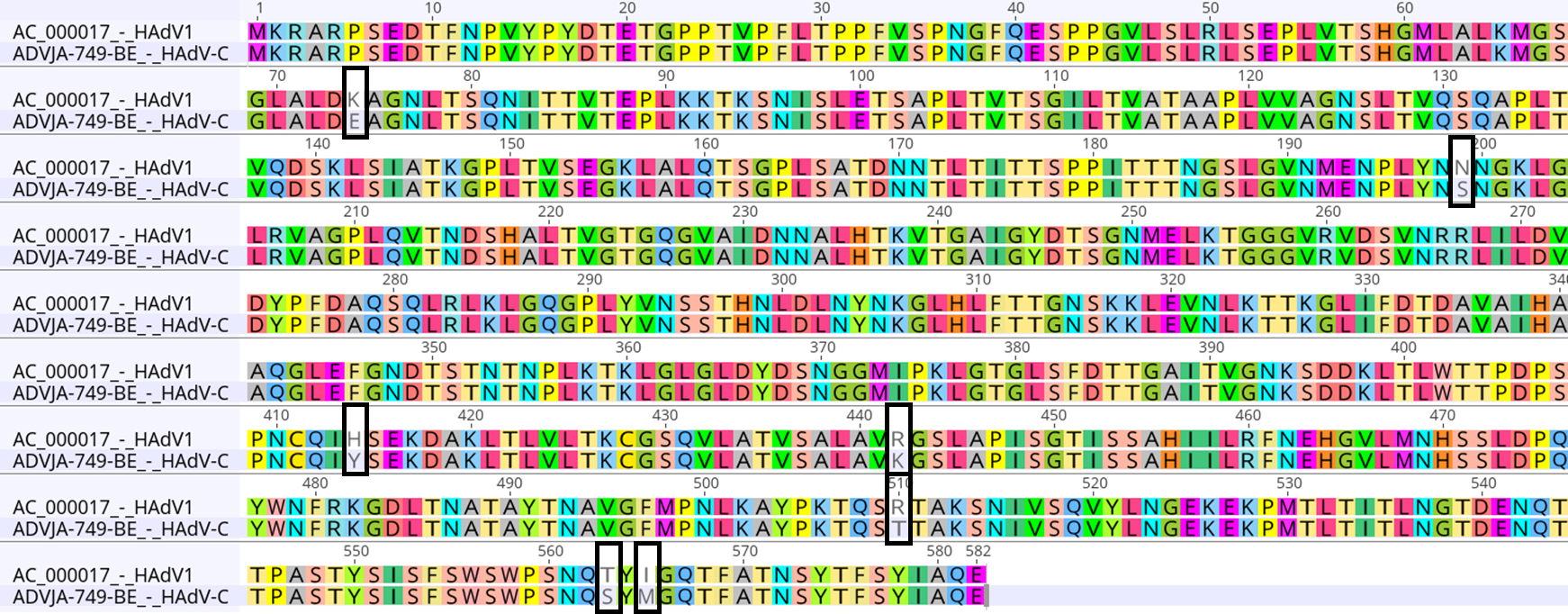


**Figure S3.** Pairwise amino acid alignment of the potentially novel HAdV-C (ADVJA-749-BE) with the suggested parent fiber prototype sequence strain HAdV1 (AC_000017.1). The name of the HAdV sequences used in the alignment analysis is indicated on the left-hand side of the figure. Labelling indicates isolate names or accession number (for prototype strains), followed by HAdV genotype/species classification. The black rectangle boxes indicated positions of amino acid differences between the potentially novel HAdV-C (ADVJA-749-BE) and the parent hexon protype sequence strains
